# Identification, Localization in the Central Nervous System and Novel Myostimulatory Effect of Allatostatins in *Tenebrio molitor* Beetle

**DOI:** 10.3390/ijms21103510

**Published:** 2020-05-15

**Authors:** Jan Lubawy, Paweł Marciniak, Grzegorz Rosiński

**Affiliations:** Department of Animal Physiology and Development, Institute of Experimental Biology, Faculty of Biology, Adam Mickiewicz University in Poznań, Uniwersytetu Poznańskiego 6 Str., 61-614 Poznań, Poland; pmarcin@amu.edu.pl (P.M.); rosin@amu.edu.pl (G.R.)

**Keywords:** *Tenebrio molitor*, neuropeptides, allatostatins, myotropic activity, MIP/AST, PISCF/AST, immunolocalization

## Abstract

Allatostatins (ASTs) are pleiotropic insect neuropeptides that are potent myoinhibitors of muscle contractions. In this study, we identified and immunolocalized peptides from the MIP/AST and PISCF/AST families in the nervous system of a model beetle, *Tenebrio molitor*. Neurons containing MIPs were immunolocalized in the brains of adults and the ventral nerve cords of larvae, pupae and imagines of this species as well as in the retrocerebral complex. PISCFs were immunolocalized in the ventral nerve cord of all stages as well as the brain of the adult beetle. Faint signals were also observed in the *corpus allatum* but not in the *corpus cardiacum*. The results allowed us to deduce the sequences of three neuropeptides belonging to MIP/ASTs, Tenmo-MIP4—NWGQFGXWa, Tenmo-MIP5—SKWDNFRGSWa and Tenmo-MIP6—EPAWSNLKGIWa, and one peptide from the PISCF/AST family, QSRYXQCYFNPISCX. Furthermore, we showed for the first time myostimulatory action of endogenous MIP/ASTs. Tenmo-MIP5 caused dose-dependent stimulation of the contractile activity of the beetle oviduct muscles, showing a sigmoidal curve up to 81.20% at the 10^−8^ M concentration, and the EC_50_ value for the myostimulatory effect of this peptide was 8.50 × 10^−12^ M. This is the first report of myostimulatory action of an endogenous myoinhibitory peptide in insect muscles.

## 1. Introduction

One of the crucial types of compounds in the nervous system of living organisms are neuropeptides. They play a fundamental role in the control of physiological processes, and they constitute one of the most diverse groups of signaling molecules in terms of function [1,2,3]. They can act as neurohormones, neuromodulators and neurotransmitters [4,5]. Together with the endocrine system, the nervous system regulates the functioning of insects and maintains homeostasis in a process known as neuroendocrine integration [6]. The neurons that produce neurohormones are known as neurosecretory cells (NSCs), and in insects, they are clustered in groups located in the protocerebrum, *pars intercerebralis* (PI), *pars lateralis* (PL) and tritocerebrum [7]. NSCs are also located in the subesophageal and ventral ganglia. The PI/PL project their axons towards the peripheral targets of the paired neurohemal *corpora cardiaca* (CC) and *corpora allata* (CA) organs, which are located on both sides of the proximal end of the aorta in beetles and form the retrocerebral complex [8].

Among insect neuropeptides, one of the largest groups is allatostatins (ASTs). ASTs have been so far identified in insect orders, such as crickets, termites, stick insects, moths, flies, cockroaches, and beetles [4,5,9]. Three separate families can be recognized among the ASTs, and although they vary structurally, they are functionally connected by inhibiting the activity of CA [10,11,12]. Although the ASTs were grouped into one large family due to their activity on the synthesis of juvenile hormone (JH), many studies have shown that this might not be their main role in the insect body [13,14]. ASTs show pleiotropic activity affecting the vitellogenesis [15,16], synthesis of digestive enzymes [17], and visceral muscle contractions [18]. For the first time allatostatins were divided into separate families by Lorenz in 1995. He recognized three families named A-, B- and C-type allatostatins [19]. Nowadays, the proposed terminology is however considered inaccurate, since it does not refer to the sequence of amino acid chains or the physiological actions of these neuropeptides. Coast and Schooley [10] tried to standardize the classification of all insect neuropeptides, with ASTs among them. They changed the name of A-type allatostatins to FGL/ASTs because of the presence of specific amino acid sequence (FGL) at the *C*-terminus. Similarly, the C-type should be referred to as PISF/ASTs because of the presence of an uncommon PISCF-OH sequence at the *C*-terminus. For the B-type allatostatins the Authors proposed the MIP/ASTs name due to the myoinhibitory action of peptides from this family on insect muscles [10]. They also proposed to construct the names of insect peptides by combining first three letters of generic name and first two letters of specific epithet of a species from which the peptide comes from. Hence, for example MIP peptides from *Tribolium castaneum* will be denoted Trica-MIP, whereas ones from *Tenebrio molitor*—Tenmo-MIP [10].

In most insects, ASTs generally are thought to be potent myoinhibitors [8,10]. FGL/ASTs are responsible for the regulation of the gut muscle and heart contractile activity [20,21]; MIP/ASTs play a myoinhibitory role in the ovary and hindgut muscles [18,22,23,24] and PISCF/ASTs are potent myoinhibitors of the foregut and heart contractions [25,26] and regulate the circadian rhythm, specifically acting as intraclock signaling molecules in flies [27]. Due to the fact that their synthesis is not limited to brain cells, but can also be found in peripheral tissues such as midgut and they play an inhibitory role, it is thought that ASTs are analogues of somatostatin in vertebrates [28,29]. Moreover, AST receptors are most closely related to the somatostatin/galanin/opioid receptor family [30,31]. Due to that fact, and the resemblance of the neuro-endocrine regulation mechanisms of insects to those of vertebrates, insects might be utilized in comparative research as model organisms [6,32,33,34].

Although some research on ASTs has been performed, especially in *Drosophila* and cockroaches, little is known about their physiological activity and myoactivity in the largest insect order—Coleoptera. To date, the only studies on this issue have been conducted by Audsley et al. [13], who reported that PISCF/ASTs do not affect the hindgut and oviduct muscles in *Tribolium castaneum*, and our previous studies. We showed that exogenous allatostatins inhibit hindgut contractions only at very high concentrations [8] or may even act as myostimulators when peptide from cricket is tested on beetle [35].

In this paper, we focus on identification on mRNA level and localization of allatostatins in the nervous system of the *Tenebrio molitor* beetle in all postembryonic developmental stages, and we show for the first time (to our knowledge) that endogenous ASTs may act as myostimulators in the insect body. It is currently thought that these peptides show stimulatory effects only in lower animals such as Annelida and Cnidaria [36,37]. However, the obtained results regarding myostimulatory activity are supported by the presence of neuropeptides in the nervous system of the beetle.

## 2. Results

### 2.1. mRNA Identification

**Figure 1:**
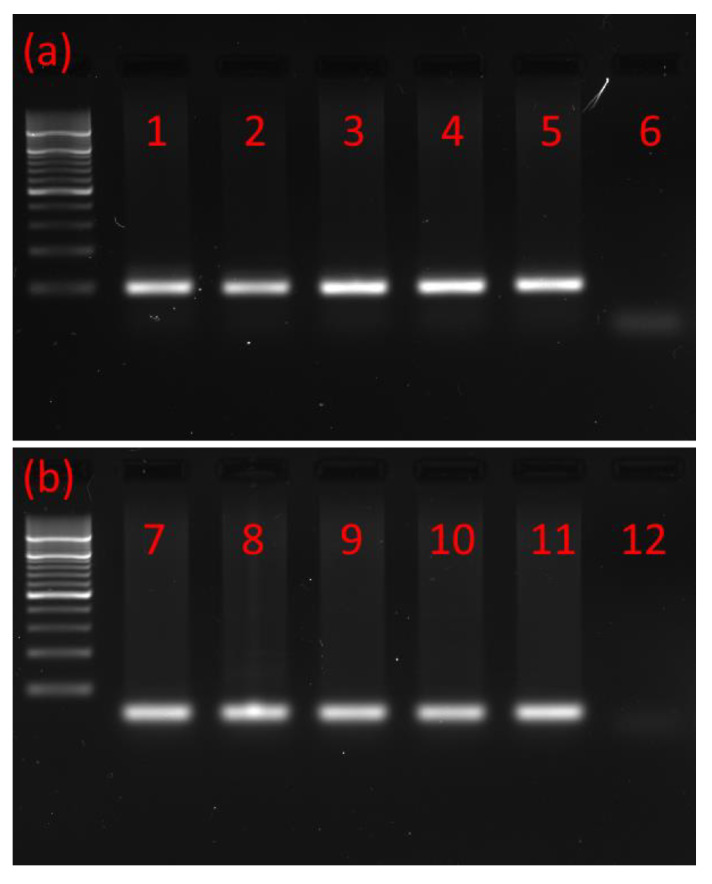
Typical agarose gel from gel electrophoresis of RT-PCR products. (**a**) PCR products with a mass of ≈200 bp showing that MIP/AST is present in the (1) adult brain, (2) adult CC/CA, (3) adult VNC, (4) pupal VNC, (5) larval VNC and (6) negative control. (**b**) PCR products with a mass of ≈100 bp showing that PISCF/AST is present in the (7) adult brain, (8) adult CC/CA, (9) adult VNC, (10) pupal VNC, (11) larval VNC and (12) negative control. CC/CA—*corpus cardiacum/corpus allatum*, VNC—ventral nerve cord. All lanes are from a single gel image, cropped for clarity and conciseness. The original gel is provided in “Supplementary Figure S1”.

Using RNA isolated from the brains and retrocerebral complexes *corpus cardiacum/corpus allatum* (CC/CA) of adults and ventral nerve cords (VNCs) of postembryonic developmental stages of the *T. molitor* beetle and primers based on *T. castaneum* MIP/AST and PISCF/AST prehormones, the cDNAs encoding part of the MIP/AST and PISCF/AST were isolated and sequenced by reverse transcription PCR. The cDNA was amplified using forward and reverse primers targeting the 3′ and 5′ ends of the predicted coding sequence. Open reading frames of 169 and 74 base pairs were detected (Figure 1a,b) encoding proteins of 55 and 27 amino acids for MIP/AST and PISCF/AST, respectively.

**Figure 2:**
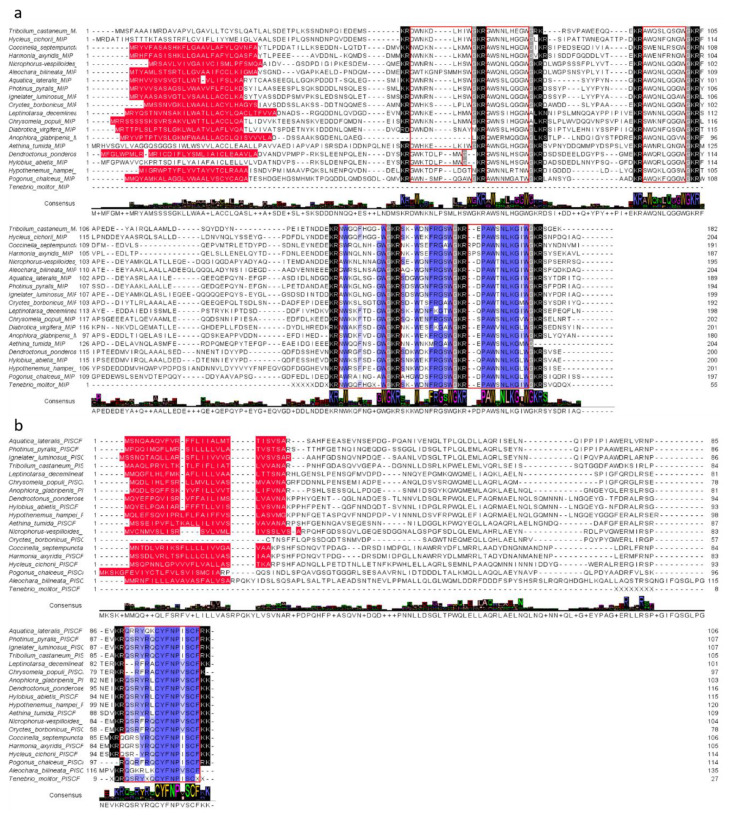
Alignment of putative MIP/AST (**a**) and PISCF/AST (**b**) sequences in beetle species and their consensus sequences with *T. molitor* identified sequences in this study. The blue color indicates similarities in the amino acid sequence. Red color—signal peptides at N-termini; black indication—endopeptidase cleavage sites; gray—amidation sites; red frames = the other deduced MIP isoforms.

The identified sequence of Tenmo-MIP 5 and two other MIP/ASTs (Tenmo-MIP 4—NWGQFGXWa, Tenmo-MIP 5—SKWDNFRGSWa and Tenmo-MIP 6—EPAWSNLKGIWa) was identical to that of *T. castaneum.* The partial sequence of the MIP/AST prehormone encodes 3 myoinhibitory peptides in *T. molitor*, being identical to those of *T. castaneum’*, which may suggest the presence of 6 myoinhibitory peptides in this species of beetle as well (Figure 2a). The identified sequence of Tenmo-PISCF also seems to be identical to that of Trica-PISCF (QSRYXQCYFNPISCX). Although we have not been able to identify the amino acid residues at positions 5 and 15 of the peptide, due to the high conservation of peptides from this family, looking at the consensus sequence it can be assumed with a very high probability that the last amino acid of this sequence is phenylalanine, whereas the fifth one is arginine (Figure 2b). These results indicate that both of these neuropeptide families—MIP/AST and PISCF/AST—are conserved among beetles and are also present in *T. molitor.*

### 2.2. Immunolocalization

**Figure 3:**
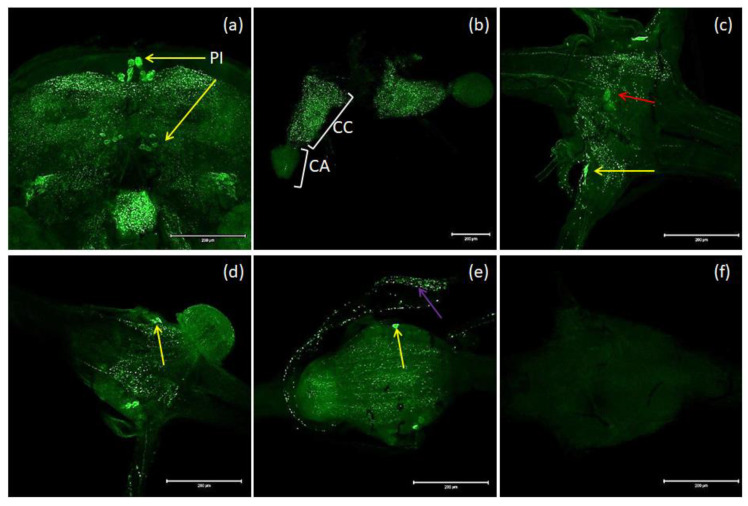
Examples of images from confocal microscopy depicting the location of MIP/AST-reactive neurons in the brain (**a**) and CC/CA (**b**) of adults as well as the VNCs of the larvae (**c**), pupae (**d**) and adults (**e**) of *T. molitor* beetles using antibodies against Drome-MIP. Control image (**f**). The selected scale is 200 μm. The yellow arrows indicate nerve cell bodies, red arrow–perisympathetic organ (PSO) and violet arrow—varicosities in axonal transport. PI—*pars intercerebralis*, CC/CA—*corpus cardiacum/corpus allatum*, VNC—ventral nerve cord.

The nomenclature of insect brain regions was based on that presented by Ito, et al. [38]. Antibodies against MIP/ASTs bind to structures in the brain, CC and VNC of *T. molitor* (Figure 3a–e). We observed six collaterally placed large perikaryons in superior neuropils. The somas of these cells form characteristic shapes in the middle of the superior middle protocerebrum (SMP) in the *pars intercerebralis* (Figure 3a). We also observed immunolabeled varicosities across whole SMP and the calyx and peduncle of mushroom bodies. Neurons containing MIP/ASTs were also identified in the central complex (up to 40–50 small perikaryons). In this part of the brain, MIP-positive cells were mostly situated in the fan-shaped body (FB). Moreover, in inferior neuropils, most likely near the inferior clamp, few labeled perikaryons were detected. Furthermore, immunolabeled fibers and varicosities were observed in the distal part of the tritocerebrum. In the VNC, pairs of bilaterally located neurons were labeled in most of the abdominal ganglia in all studied stages. In pupae, two pairs of neuronal somas could be observed, while in larvae and adults, one pair could be observed (Figure 3c–e). In the larval stage, MIP-positive cells were also found in the perisympathetic organs located in the middle of the VNC (Figure 3c). MIP-positive varicosities were detected in the connectives between ganglia and in segmental peripheral nerves (Figure 3c–e). Immunolabeled fibers and varicosities were also observed in the *corpora cardiaca*; however, in the *corpora allata*, there was almost no signal (Figure 3b).

**Figure 4:**
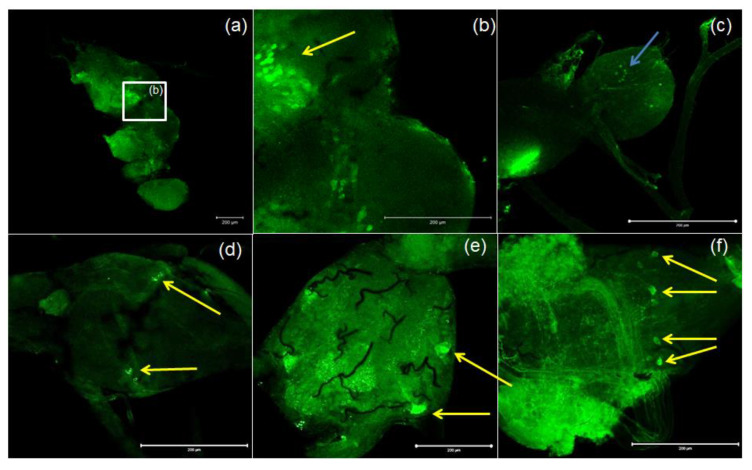
Examples of images from confocal microscopy depicting the location of PISCF/AST-reactive neurons in the brain (**a**,**b**) and CC/CA (**c**) of adult as well as the VNC of the larvae (**d**), pupae (**e**) and adults (**f**) of *T. molitor* beetle using antibodies against Manse-PISCF. The selected scale is 200 μm. The yellow arrows indicate the nerve cell bodies, and the blue arrows show signals present in the CA. CC/CA—*corpus cardiacum/corpus allatum*, VNC—ventral nerve cord.

**Figure 5:**
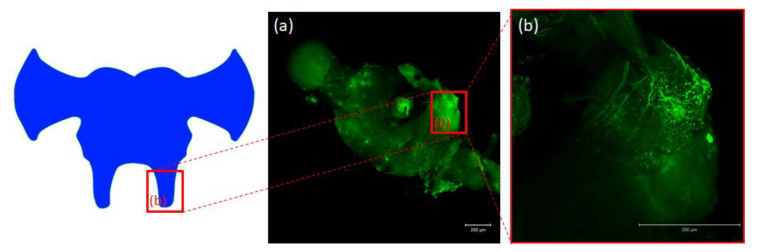
Schematic representation and images from confocal microscopy (**a**) of insect brain with immunofluorescent signals from anti-Manse-AST C in tritocerebrum (**b**), tritocerebrum with varicosities, fibers and somas containing Tenmo-PISCF. The selected scale is 200 μm.

Antibodies against PISCF/ASTs bound to a number of somas in the protocerebrum in regions of the medial lobe (ML), which is a component of the mushroom bodies (MB) in the beetle’s brain (Figure 4b). PISCF-positive varicosities and cells could also be detected in the distal part of the tritocerebrum (Figure 5). When an anti-Manse-AST C antibody was used in the retrocerebral complex of adult *T. molitor*, a weak signal was observed in the glands of the *corpora allata*, while the *corpora cardiaca* glands were not marked in this complex (Figure 4c). Structures immunolabeled with these antibodies were observed in the VNC of *T. molitor* at all studied stages (Figure 4d–f). The signals for PISCF/AST were also detected in the terminal abdominal (TAG) and thoracic ganglia in the VNC. A double pair of laterally located neuron somas was identified in the TAG of pupae (Figure 4d). We also identified two pairs of perikaryons in the TAG of adults; however, they were located posteriorly (Figure 4e). The positive signals observed for the tested peptides, indicate that MIP/ASTs and PISCF/ASTs are endogenously expressed in nervous tissues of *T. molitor* and can be localized to multiple neuropils.

### 2.3. Oviduct Contractions

**Figure 6:**
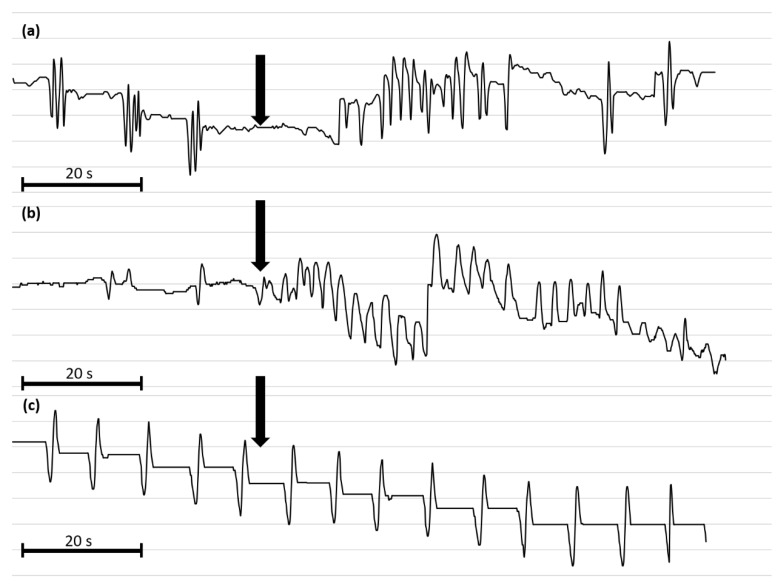
Representative myograms of *T. molitor* oviduct contractile activity after the application of Tenmo-MIP 5 at 10^−10^ mol/L (**a**), proctolin at 10^−5^ mol/L (**b**) and physiological saline (**c**). The arrows show the time of neuropeptide application. The scale bars indicate 20 s of video recording.

**Figure 7:**
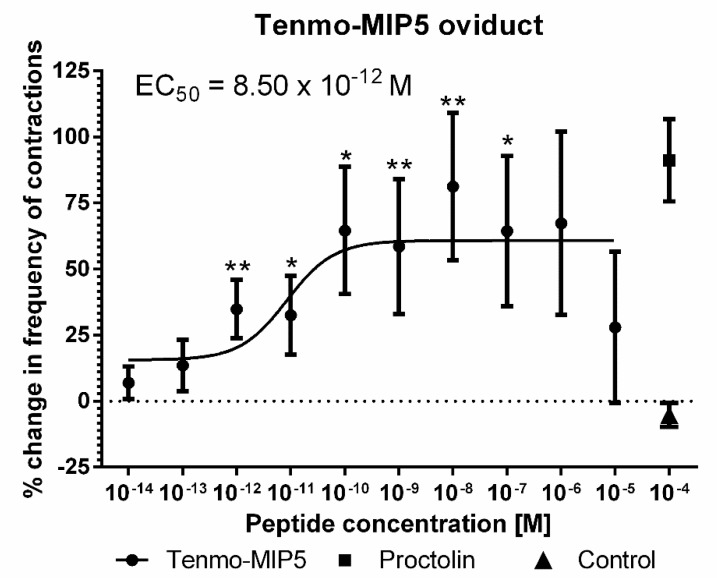
Changes in the oviduct contractile activity of *T. molitor* after the application of Tenmo-MIP 5 compared to the control. Means ± SEM are given for *n* ≥ 15. Significant differences from the control (saline application) are indicated by * *p* ≤ 0.05, ** *p* ≤ 0.01, (one-way ANOVA). Proctolin, a potent myostimulator, was used as a positive control.

The effects of synthetic Tenmo-MIP 5 on the oviduct contractile activity of *T. molitor* was tested in the oviduct bioassay (Figure 6). Since the Tenmo-PISCF seems to be identical to Trica-PISCF tested previously [35], we omitted this peptide in our studies. Under constant superfusion with physiological saline in control conditions, the mean frequency of oviduct contractions was 8.42 ± 6.03 contractions per minute. The application of an additional 10 µL of physiological saline (PS) did not cause any significant changes in the activity of the oviducts. The peptide caused dose-dependent stimulation of contractions in the oviduct muscles of the beetle, showing a sigmoidal curve (Figure 7). The highest stimulation (81.20%) was observed at a 10^−8^ M concentration (*t =* 3.284; *p* = 0.005), and the calculated EC_50_ value was 8.50 × 10^−12^ M (Figure 7). Similar to Trica-PISCF, Tenmo-MIP 5 showed a myostimulatory effect over the whole range of the tested concentrations in *T. molitor*. However, the effect of Tenmo-MIP 5 was more profound than that of Trica-PISCF [35]. The presented results indicate that allatostatins may play myostimulatory role in *T. molitor* body.

## 3. Discussion

Here, we report the identification and immunolocalization of peptides from two allatostatin families, MIP/AST and PISCF/AST, in the nervous system of *T. molitor* beetle. We found high homology between identified peptides and other beetle MIP and PISCF family peptides (Figure 2). We also show, for the first time to our knowledge, the myostimulatory effect of endogenous MIP/AST–Tenmo-MIP 5 on the contractile activity of visceral muscles in insects. It was previously thought that these peptides may act in a myostimulatory manner but only in lower organisms such as annelids or cnidaria and not in insects [36,37].

Myoinhibitory peptides belong to the allatostatin family, whose main role is the inhibition of synthesis and secretion of juvenile hormone [9,39]. Nevertheless, MIP/ASTs show a pleiotropic mode of action, including inhibition of food intake and the contractile activity of visceral muscles [9]. Additionally, fibers containing MIP/ASTs were found to innervate visceral organs [4,9,18,22,39,40,41]. MIP peptides are characterized by amidation at the *C*-termini and the presence of W at the *C*-terminus at positions 1 and 8 (W(X_6_)Wa) [9,10]. Although this sequence is characteristic of MIP/ASTs, some representatives of this family possess a W(X_7-8_)Wa sequence [18]. The first peptide from MIP/AST family, Grybi-MIP 1 (GWQDLNGGWa), was identified in 1991 from a cricket—*Gryllus bimaculatus* [42], and all of the MIP/AST peptides are synthetized from one precursor that generates three to 11 structurally related peptides [43,44]. Taking the above into consideration, the aa sequence of peptides from MIP family is characterized by high variation in insects. In our research, we were able to identify three isoforms, which were identical to those of *T. castaneum.* This result was not surprising considering that these insects belong to the same family. The recently published papers by Veenstra [45] and Pandit, et al. [46] show that the MIP/AST gene encodes six isoforms in *T. molitor*. However, they conducted in silico neuropeptidomic studies, which seems to be contrary to each other in some aspects, hence further studies are needed to identify three remaining MIP peptides found in this insect body. In contrast to other known allatostatins, PISCF genes encode one biological peptide, which seems to be highly conserved. Most of the identified sequences are predicted to generate peptides homologous to those of *M. sexta* or with small differences in structure. For example, the peptide predicted and identified from *T. castaneum* differs in two positions from the sequence of *Manduca sexta* [28,47]. Tenmo-PISCF also seems to be identical to Trica-PISCF. Although we have not been able to identify the whole amino acid sequence of the peptide, it can be assumed almost with certainty that the last amino acid residue in the sequence is phenylalanine and that the other one is either arginine or serine [28].

MIP-immunoreactive cells have been identified in the nervous system and other tissues of many insects such as *M. sexta* [22], *Rhodnius prolixus* [18], *Periplaneta americana* [48], and *Leucophaea maderae* [49]. In beetles, researchers have identified MIP/ASTs in the brain of *T. castaneum* [50] and *Zophobas atratus* [51] using mass spectrometry or localized them to the brain and VNC of *Leptinotarsa decemlineata* [52] and *Nicrophorus vespilloides* [8]. In the brain of *T. molitor* we observed comparable localization of MIP-positive neurons as previously in another beetle, *N. vespilloides* [8], and the cockroaches *L. maderae* and *Periplaneta americana* [48,49]. These allatostatins are transported to the CC/CA by *nervi corpori cardiaci*, [48,49,53]. We also observed perikaryons containing MIPs in the central complex in FB. Almost identical localization of these peptides was also observed in *N. vespilloides* and *L. maderae*. However, in the latter, immune-labeled cells were observed across the whole central complex [8,49]. These results suggest that MIP/ASTs may be responsible for the regulation of locomotory activity and food intake [18], and in beetles, it may be expressed in a more middle area of the brain [8]. As observed in two other beetles, *N. vespilloides* [8] and *L. decemlineata* [52], MIP-positive fibers as well as varicosities were identified in the SMP and the calyx part of the MB in *T. molitor*. This may show a role of MIP/AST as a neuromodulator in nervous system [52]. Like in other research conducted on different insects, we identified a large number of fibers and varicosities in CC; however, the signal in CA was very faint [8,20,22]. We identified MIP-positive cells in all ganglia in the VNCs of all three developmental stages of *T. molitor*. Similar results to ours regarding the bilateral arrangement of MIP-positive cells in the VNC have been reported in the species *N. vespilloides* and *R. prolixus* [8,18]. In the case of *T. molitor* larvae, we also observed stained cells in the middle part of the abdominal ganglia. These cells may belong to perisympathetic organs (PSOs), which have also been shown to possess MIP/ASTs in the cockroach *P. americana* [48] and the stick insect *Carausius morosus* [54]. Moreover, we observed axons labeled with MIP antisera in the connectives between ganglia as well as in segmental peripheral nerves.

In the *T. castaneum* nervous system, a wide occurrence of PISCF/ASTs has been found, and the neurons synthesizing these peptides are present (although with weak positive reactions) in both the medial and lateral cells of the brain. In the *T. castaneum corpus cardiacum*, the Trica-PISCF peptide is present, which was confirmed by immunocytochemistry, but it is missing in the *corpora allata* of this insect [13]. The authors of this study suggest that the peptides from the PISCF family do not participate in the regulation of JH synthesis in *T. castaneum*. However, in a 3-day-old *T. molitor* female, Trica-PISCF suppressed the synthesis of JH, while in 7-day-old females, the opposite effect was observed, and the magnitude of the induced reaction was the same as for allatotropin, increasing the level of this hormone [55]. The results of Audsley, et al. [13] research also seem to contradict the results of the experiments conducted in this research. In the *corpus allatum* of *T. molitor*, there was a weak positive signal for allatostatin-like peptides from the PISCF/AST family, whereas there was no immunoreactive reaction in CC, as was the case for *T. castaneum* [13]. Further studies involving the measurement of JH biosynthesis in the CA of *T. molitor* after the application of PISCF/AST are required to dispel doubts about the role of these peptides in JH synthesis. As mentioned above, in the brain of *T. castaneum*, antisera against PISCF/ASTs weakly bind to median and lateral neurosecretory cells of the brain. In our study, we found that PISCF/AST-positive cells are widely present in the ML as well as the distal part of the tritocerebrum. The difference in occurrence could be related to the role that allatostatins play in various insect species. Audsley, et al. [13] stated that in tenebrionidae, ASTs participate mainly in the stimulation of the proteases’ secretion since the general activity of these enzymes rises when PISCF/ASTs are injected. It is possible that isolation of CNS while or immediately after food intake could give different results. On the other hand, since PISCF/ASTs show either neurotransmitter or neuromodulator action, this peptide in the *T. molitor* brain can act as a signaling molecule regulating circadian rhythm, as observed in *D. melanogaster* [27]. The presence of PISCF/AST in the ML, the part of the mushroom bodies (MB) that plays a role in the processing of visual input [56], may further support this claim. The results obtained by Audsley et al. [13] regarding the location of an allatostatin from the PISCF/AST family in the VNC of *T. castaneum* are similar to those obtained in the present study for *T. molitor*. In the VNC of *T. castaneum*, a strong immunopositive reaction for Trica-PISCF was mainly detected in the TAG. However, in the VNC of *T. molitor*, in addition to neurons in the TAG, cells were also labeled in other ganglia, both in the larva and the pupa. Considering the presence of PISCF/AST allatostatin receptors in the ovaries and the fat body of beetles as well as a role in increasing protease activity [13], the effect of Trica-PISCF on oviduct and hindgut muscles, which we have shown previously [35], and the presence of these allatostatins in the ganglia of the VNC, especially in the TAGs, which endings of neurons reach the ovaries and gut, one can infer that these peptides may play an important role in controlling the growth and development of oocytes during the gonadotropic cycle and digestion process in the *T. molitor* beetle. In insects like cockroaches and flies, allatostatins are also responsible for the increase in the activity of digestive enzymes as well as regulation of food intake [17,57]. The presence of Trica-PISCF in endocrine cells, the expression of its receptor in the gut of *T. castaneum* [13] and the presence in the VNC of *T.* molitor suggest that this peptide is functionally significant and needed to properly regulate the activity of digestive tract in beetles.

The most interesting findings of this study were the results of the muscle contraction bioassay. Allatostatins belonging to the MIP/AST are described by their inhibitory action on the insect visceral muscles [24,58]. Due to these properties, it has been thought that these allatostatins present the potential to inhibit movement of eggs in the oviduct. However, the results of our studies, presented both here and earlier [35], seem to oppose this, indicating that some allatostatins in *T. molitor* may show a myostimulatory effect. The conducted research showed that Tenmo-MIP 5 acts in a different manner towards oviduct muscles than has previously been shown for MIP/ASTs in homologous systems [58]. Tenmo-MIP 5 increases the contractile activity of the common oviduct in *T. molitor.* Most importantly, in contrast to the results of heterologous assays [35], the presented effect was dose dependent, showing a typical sigmoidal curve and a low EC_50_ value. Overall, as increases in contractions and the duration of the effect associated with the dose applied are considered characteristic of the receptor-mediated response, the observed dose-dependent effect suggests the physiological nature of the process. The effect produced by this allatostatin, which differs from the rest of this family, is similar to that of Rhopr-MS [24]. Myosupressins, to which Rhopr-MS belongs, are strong mioinhibitors of insect visceral muscles [59], but surprisingly, this peptide increases the contractile activity of the oviduct in *R. prolixus* [24]. We obtained a similar myostimulatory effect for allatostatins Grybi-MIP 1, however, with no dose-dependency, and Trica-PISCF [35], the latter of which was shown here to be identical to Tenmo-PISCF. The unique properties of neuropeptides, whose myotropic effects are well described, are not only the case for MS. As an example, proctolin a well-established cardio and myostimulator, shows no effects on the hearts of *Stomoxys calcitrans* and *M. sexta* [60]. Although, in another beetle, *N. vespilloides* Trica-MIP 5 causes a decrease in gut muscles activity, this effect was noticable only in higher concentrations (10^−5^ M) than those at which neuropeptides usually act in a physiological state. The myostimulatory effects of both MIP/AST and PISCF/AST have been previously shown in lower organisms. Williams, et al. [36] showed that activation of MIP/AST signaling by the addition of a synthetic peptide causes an increase in gut peristalsis and in frequency of extensions of pharynx in the annelid *Platynereis dumerilii.* Alzugaray, et al. [37] showed that high doses of PISCF/AST induce a particular form of contractions that bear a resemblance to the peristaltic waves that are present in the digestive system of most organisms [36,37].

In summary, our results clearly show similarity between the localization of allatostatins in the central nervous system and their sequences between beetle species. However, the results of the contractile bioassay provide a novel point of view regarding the probable role of these neuropeptides in the insect body. Unfortunately, at this point, it is difficult to explain the differences in the myostimulatory nature of action of the tested peptides and myoinhibitory actions noted in other insects. The existence of several isoforms of the same peptide suggests that for a proper mode of action of one isoform in a physiological state, the presence of the others is necessary, as they may act synergistically or antagonistically. It could also be possible that during evolution in different phyletic lineages, the signs of regulation switched in beetles.

Further research is needed to identify specific allatostatin receptors, which are insect GPCRs, in *T. molitor* [9]. They play a role during oogenesis, vitellogenesis and muscle contraction. It has also been shown that the administration of ASTs and their analogs with food reduces the survival of pest insects [61] Thus, the linkage of the role of AST receptors and ASTs in the key developmental and metabolic processes makes these peptides a prospective targets for pesticide design. However, these issues require further studies, especially comparative morphological and molecular studies, in a broader range of taxa.

## 4. Materials and Methods 

### 4.1. Insects

The adults, pupae and larvae of *T. molitor* were obtained from a colony maintained in the Department of Animal Physiology and Development at Adam Mickiewicz University, Poznań, Poland. The beetles were reared as previously described [35,62]. For the experiments, 4-weak-old adults and 3-day-old pupae were used. Because the number of larval instars in this beetle varies from 10 to 16 and the duration of each stadium may differ [63], larvae of the same body weight (140 ± 10 mg) were chosen for the experiments.

### 4.2. Isolation of mRNA and Generation of cDNA

Whole brains, CC/CAs and VNCs from three stages of *T. molitor* were dissected in physiological saline (PS) (274 mM NaCl, 19 mM KCl, 9 mM CaCl_2_, 5 mM glucose, and 5 mM HEPES, pH 7.0, Sigma-Aldrich, Poznań, Poland). Total RNA was extracted from ten samples of each tissue (10× brains, 10× CC/CAs and 10× VNCs) using the Insect RNA MicroPrep™Kit (Zymo Research Corp., Irvine, CA, USA) according to the manufacturer’s protocols. The protocol included the in-column DNase I treatment to remove traces of gDNA and prevent the contamination of the RNA samples. ReverAid™ Reverse Transcriptase (Fermentas, Waltham, MA, USA) was then used to generate cDNA for PCR according to the manufacturer’s protocols.

A Bio-Rad T100 thermocycler (Bio-Rad, Hercules, CA, USA) was employed for PCR using the following cycling conditions: 95 °C for 3 min, followed by 34 cycles of 95 °C for 30 s, 56 °C for 30 s and 72 °C for 60 s. The PCR products were separated using 2% agarose gels in TEA buffer and visualized using ethidium bromide. Gel products were purified using the Zymoclean™ Gel DNA Recovery Kit (Zymo Research Corp., Irvine, CA, USA) and sequenced with BigDye Terminator v3.1 in an ABI Prism 3130XL Analyzer (Applied Biosystems, Foster City, CA, USA) according to the manufacturer’s protocols.

### 4.3. Primer Design

We used the *T. castaneum* sequences for MIP/AST (accession numbers: NM_001143730.1) and PISCF/AST (accession number: FJ415748.1) for primer design. Forward and reverse PCR primers were designed using Primer-BLAST (https://www.ncbi.nlm.nih.gov/tools/primer-blast/) developed at NCBI, which uses Primer3 to design PCR primers and then BLAST and global alignment algorithms to screen primers against user-selected databases. Gene-specific primers with a 20 nt length, 50–60% GC content and 60 °C melting point were selected. The PCR primers were custom synthesized at the Institute of Biochemistry and Biophysics of the Polish Academy of Sciences (http://oligo.ibb.waw.pl/English/, Warsaw, Poland). The primers were reconstituted in RNase/DNase-free water to a concentration of 100 pmol/μL. The primers used to generate the partial sequences of the Tenmo-MIP and Tenmo-PISCF prehormones are listed in Supplementary Table S1.

### 4.4. BLAST Search

A BLAST analysis based on the available sequences of *Aethina tumida* (AN: PRJNA361278 and PRJNA256171), *Aleochara bilineata* (AN: PRJNA378164), *Anoplophora glabripennis* (AN: PRJNA348318), *Aquatica lateralis* (AN: PRJDB6460), *Chrysomela populi* (AN: PRJNA212154), *Coccinella septempunctata* (AN: PRJDB7050), *Dendroctonus ponderosae* (AN: PRJNA162621 and PRJNA360270), *Diabrotica virgifera* (AN: PRJEB28633 and PRJNA206802), *Harmonia axyridis* (AN: PRJDB3183), *Hycleus cichorii* (AN: PRJNA349771), *Hypothenemus hampei* (AN: PRJNA279497), *Hylobius abietis* (AN: PRJNA435680), *Ignelater luminosus* (AN: PRJNA418169), *Leptinotarsa decemlineata* (AN: PRJNA420356), *Nicrophorus vespilloides* (AN: PRJNA339573), *Oryctes borbonicus* (AN: PRJNA293509), *Photinus pyralis (AN:* PRJNA378805), *Pogonus chalceus* (AN: PRJNA381601) and *Tribolium castaneum* (AN: PRJNA15718) were performed. The identified nucleotide sequences were translated to amino acid sequences using the ExPASy translate tool (https://web.expasy.org/translate/). For the alignment of neuropeptide amino acid sequences, JalView 2.10.1 software was used [64]. The signal peptides were predicted using SignalP [65] as implemented on the Web (http://www.cbs.dtu.dk/services/SignalP/).

### 4.5. Immunohistochemistry

Immunohistochemistry analysis was conducted based on the method previously described by Marciniak, et al. [62] and Urbanski, et al. [8]. Isolated brains, ventral nerve cords and retrocerebral complexes of *T. molitor* were fixed in fresh 2% paraformaldehyde (Sigma-Aldrich, Poznań, Poland) in phosphate-buffered saline (PBS; Sigma-Aldrich, Poznań, Poland) (pH = 7.2) for 24 h Then, the samples were washed in PBS and incubated at 4 °C in 4% Triton X-100 (Sigma-Aldrich, Taufkirchen, Germany). Next, isolated structures were kept overnight at 4 °C in 4% Triton-X 100, 2% normal goat serum (NGS; Jackson ImmunoResearch Lab., West Grove, PA, USA), and 2% bovine serum albumin (BSA; Sigma-Aldrich, Poznań, Poland) to enhance antibody penetration and reduce nonspecific binding. The primary antibody (1:500 dilution) was next administered in mixture of PBS with 0.4% Triton-X 100, 2% NGS, and 2% BSA. For identification of the tested neuropeptides, previously tested, Drome-MIP [8] and Manse-PISCF [13] antisera were used. Anti-Drome-MIP was a gift from Prof. J.A. Veenstra, and Anti-Manse-PISCF was a gift from Dr. Neil Audsley. Next the tissues were washed with cold PBS for 4–5 h, and incubated with secondary antisera for 24 h at 4 °C in darkness. The secondary antibody was fluorescein (FITC)-conjugated Affinity Pure Goat Anti-Rabbit IgG (H + L) (Jackson ImmunoResearch Lab., West Grove, PA, USA) diluted 1:200 in fresh PBS. Lastly, the samples were washed trice in cold PBS and mounted in 90% glycerol with DABCO on microscope slides. The samples were analyzed with a confocal microscope (LSM 510, Axiovert 200 M, Carl Zeiss, Germany). To exclude nonspecific binding of secondary antibodies, a control was performed in which fixed tissues were incubated in PBS with 0.4% Triton-X 100, 2% NGS, and 2% BSA without primary antisera and with primary antibodies saturated with synthetic peptides.

### 4.6. In Vitro Oviduct Bioassay

To assess the effects of Tenmo-MIP 5 on the contractile frequency of isolated oviducts, we used the videomicroscopy method, coupled with computer-based data acquisition and analysis, as previously described [35]. The oviduct with the ovaries was isolated from 4-week-old females and placed in an incubation chamber filled with 100 μL of PS under an Olympus SZX12 stereomicroscope equipped with an Olympus SC30 camera (Olympus, Tokio, Japan). Each preparation was continuously superfused with fresh PS at a flow rate of 140 μL per min in the incubation chamber. Prior to recording, each sample was stabilized for 10 min in PS. The movie was recorded for 2 min. The tested peptides were applied after 0.5 min of the recording using 10 μL Hamilton syringe (Hamilton Co., Nevada, USA) at maximal volume. Myograms were constructed from the video clips using AnTracker software ver. 1.0 (AnTracker; www.mikroskop.com.pl) after binarization. The activity of the oviduct was tested with Tenmo-MIP 5 (SKWDNFRGSWa) purchased from Creative Peptides (New York, NY, USA). The activities of the peptides are shown as the percent changes in the control frequency of the isolated organ contractions.

### 4.7. Statistical Analysis

For a statistical analysis of the obtained data, we used GraphPad software (ver. 6) (Department of Animal Physiology and Development AMU license). Before the statistical analysis, the normality of the distribution (the Shapiro-Wilk test) and the homogeneity of the variance (the Brown-Forsythe test and the Levene test) were checked. For the analysis of groups with a normal distribution, one-way ANOVA with Tukey’s post hoc test was used. To draw the dose-response curve and EC_50_ values_,_ nonlinear regression was used. Significant results were considered those with a *p*-value of *p* ≤ 0.05 (*), *p* ≤ 0.01 (**) or *p* ≤ 0.001 (***).

## Supplementary Materials

Supplementary materials can be found at https://www.mdpi.com/1422-0067/21/10/3510/s1. Figure S1: Full gel presented in the manuscript in Figure 1. Table S1: Primers used in this study.
