# Supplementary material for: Identification, Localization in the Central Nervous System and Novel Myostimulatory Effect of Allatostatins in Tenebrio molitor Beetle
Source: Int J Mol Sci. 2020 May 15;21(10):3510. doi: 10.3390/ijms21103510 (PMC7279042; doi:10.3390/ijms21103510)
Supplement: Supplementary file 1 [file ijms-21-03510-s001.pdf]

Supplementary Information for:

Identification, localization in the central nervous system and novel myostimulatory effect of allatostatins in *Tenebrio molitor* beetle

Lubawy Jan*, Marciniak Paweł, Rosiński Grzegorz

[1]Department of Animal Physiology and Development, Institute of Experimental Biology, Faculty of Biology, Adam Mickiewicz University in Poznań, Uniwersytetu Poznańskiego 6 Str., 61-614 Poznań, Poland

*Corresponding author

Jan Lubawy

j.lubawy@amu.edu.pl

Figure S1 Gel presented in the manuscript in Figure 1 is shown here (Fig. S1)

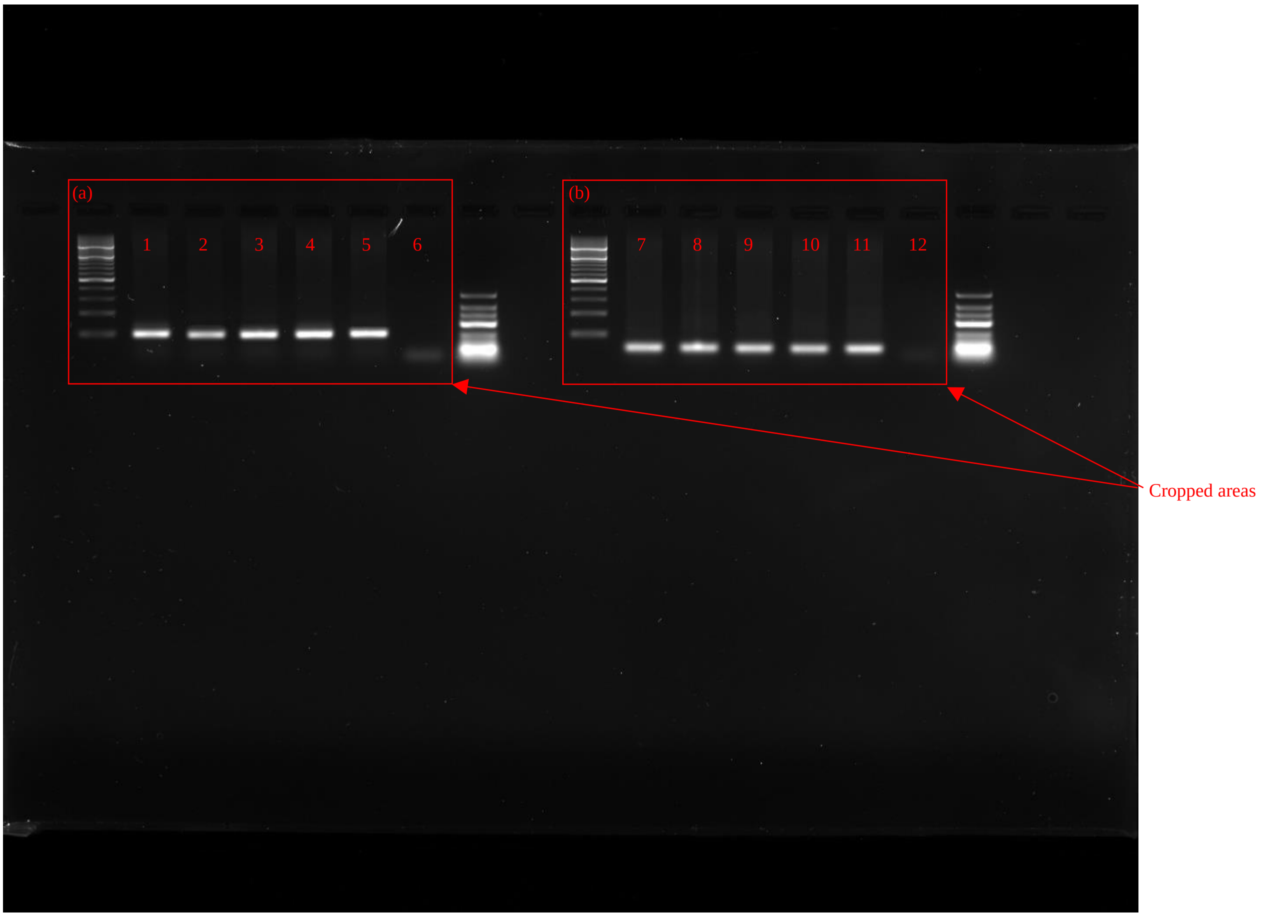

Figure S1. Agarose gel from gel electrophoresis of RT-PCR products with a mass of ≈200 bp showing MIP/AST (lines 1-5) and a mass of ≈100 bp showing PISCF/AST (lines 7-12). From left to right lines present: marker A, MIP/AST present in the adult brain, MIP/AST present in the adult CC/CA, MIP/AST present in adult VNC, MIP/AST present in pupal VNC, MIP/AST present in larval VNC, negative control, marker B, empty line, marker A, PISCF/AST present in the adult brain, PISCF/AST present in the adult CC/CA, PISCF/AST present in the adult VNC, PISCF/AST present in the pupal VNC, PISCF/AST present in the larval VNC, negative control, marker B. CC/CA – *corpus cardiacum/corpus allatum,* VNC – ventral nerve cord.

Table 1. Sequences of primers designed for Tenmo-MIP and Tenmo-PISCF used in the study

	**Tenmo-MIP**	**Tenmo-PISCF**
**Forward primer**	AAGGACTTGCACATCTGGGG	CAAACAGGGGGAGACTACGG
**Reverse primer**	GTCGTATTGGGGTTCCAGCA	CTGAAGCAGCTGATGGGGTT
